# Emotions and Reactions to the Confinement by COVID-19 of Children and Adolescents With High Abilities and Community Samples: A Mixed Methods Research Study

**DOI:** 10.3389/fpsyg.2020.585587

**Published:** 2020-11-23

**Authors:** María de los Dolores Valadez, Gabriela López-Aymes, Norma Alicia Ruvalcaba, Francisco Flores, Grecia Ortíz, Celia Rodríguez, África Borges

**Affiliations:** ^1^Institute of Psychology and Special Education, Department of Applied Psychology, University Center for Health Sciences, University of Guadalajara, Guadalajara, Mexico; ^2^Transdisciplinary Research Centre in Psychology, Autonomous University of the State of Morelos, Cuernavaca, Mexico; ^3^Doctorate Interinstitutional Psychology, Department of Applied Psychology, University of Guadalajara, Guadalajara, Mexico; ^4^Integral Development of the Family, Tlajomulco de Zúñiga, Mexico; ^5^Department of Clinical Psychology, Psychobiology and Methodology, Faculty of Health Sciences, University of La Laguna, San Cristóbal de La Laguna, Spain

**Keywords:** children, COVID-19, emotions, teenagers (adolescence), gifted students

## Abstract

The goal of this research is to know and compare the emotions and reactions to confinement due to the COVID-19 pandemic in children and adolescents with high abilities and community samples. This is a mixed study with an exploratory reach that is descriptive, and which combines survey and qualitative methodologies to examine the emotions and reactions to confinement experiences of children and adolescents aged between 5 and 14 years. An online poll was designed with 46 questions, grouped into three sections: (1) General Data, (2) Reactions to the health contingencies of COVID-19, and (3) Positive and negative emotions. This last section was included the Oros’ positive emotions questionnaire (2014) and the scales of the negative effect of the PANAS Schedule positive and negative affect on children and adolescents between 8 and 14 years old (Sandin, 2003). Data were collected online with a convenience sample. Quantitative data were analyzed with the SPSS statistics program and qualitative data with Alceste software. Among the main findings is the fact that there are no significant differences between the groups by ability in terms of reactions and emotions; however, there are differences between age groups and sex. The study discusses the implications for educational intervention in situations like the current pandemic.

## Introduction

According to the World Health Organization (World Health Organization, 2010), a pandemic is a worldwide spread of a new disease. The coronavirus SARS-CoV-2 is a new virus to which humans have no antibodies and its consequences are serious due to the ease of transmission between people, which have high contagion figures and have caused a saturation of the health system. On 11 March 2020, the WHO declared COVID-19 as a pandemic because it has affected over 180 countries, and suggested a series of actions to mitigate its effects on the community, in particular social isolation.

Quarantines or isolation, either voluntary or mandatory, are one of the preventive health measures to avoid the disease spread. A quarantine can last days, weeks, or months, depending on the way the disease spreads and its severity. Even though isolation is a measure that aims to protect physical health, it is still likely to have an impact on mental health.

Social connection is one of the most deeply rooted evolution characteristics of human beings and is strongly related to survival. On the other hand, isolation is one of the factors that can threaten a person’s state of mind. At the same time, people that feel alone or isolated have a higher probability of showing depressive symptoms (Santini et al., 2020).

A review of different studies related to quarantines has revealed that states of isolation have an important psychological impact on people (Brooks et al., 2020). It has even been shown that the conditions generated by social isolation create non-normative stressors, that increase the chance of mental problems appearing for the first time, and they even aggravate or have a manifest recurrence in pre-existing mental disorders (Caballero-Domínguez and Campo-Arias, 2020).

Social distancing has been one of the preventive recommendations during the COVID-19 pandemic. People have been asked to keep a minimum distance of up to 2 m from others to avoid contagion, and in the same way, it has been discussed that social distancing could have a direct impact on mental health (Kleinberg et al., 2020).

But, what is happening with children? Although most of the research shows that the psychological well-being and mental health of adults are affected by the current health crisis, it is important to determine to what extent it influences children, since they are vulnerable to the emotional impact of experiencing traumatic events that affect their daily lives (Bartlett et al., 2020). For this reason, it is expected that children will present emotional and behavioral changes while the contingency measures are in place. There is evidence from UNICEF (Guterres, 2020) that one of the pandemic’s effects is a rise in family levels of stress, so it is of special interest to analyze how this affects children. Various authors and institutions point out that children react to the social state of adults (Centers for Disease Control and Prevention, 2020). Hence, it is stated that the children are fine when they are in tune with the emotional states of the adults (Dalton et al., 2020) and that, while being active observers of the people around them they look at hints on how to deal with their own emotions (World Health Organization, 2020) and identify and react to the parents’ stress, or their caregivers or other members of their family and community (Bartlett et al., 2020).

An important element in the perception of emotions is the level of activation and valence, for example, in boys it has been observed that there are gender differences in emotional reactivity to girls, who tend to react more intensely to emotions related to aversion (Sharp et al., 2006). On the other hand, studies have shown that boys classify emotions similarly to adults in terms of valence; however, differences have been observed in reactivity, as boys tend to qualify their emotional states with more neutrality (Mouw et al., 2016).

In children with high ability, there is debate regarding their emotions (Francis et al., 2016). Some authors report that these children can show feelings like anxiety and depression due to their high sensibility (Silverman, 1993; Wellisch and Brown, 2012; Karpinsky et al., 2018; Armstrong et al., 2019). However, others point to the opposite, indicating that they have a good socio-emotional fit (Valadez et al., 2015; Egeland, 2019; França-Freitas et al., 2019). Meanwhile, Borges et al. (2011) say that cognitive abilities can be used to solve any problems, allowing them a similar adjustment to their fellow partners.

Another study of the emotions of children and adolescents analyzed differences by sex (Heras et al., 2016), finding contradictory results (Madden et al., 2000; Etxebarria et al., 2003; Cova et al., 2005; Prieto et al., 2008; Palazuelo et al., 2010; Heras et al., 2016; Khamis, 2018; Van der Cruijsen et al., 2019). It is said that girls have a greater emotional development; however, other studies have found that girls have higher sadness scores and boys show higher aggression scores (Etxebarria et al., 2003). For pre-teens (11–13 years old), it was pointed out that women show higher emotional distress and depression than males. This matches reported by other scholars, which found that women have higher emotional distress, like anxiety and depression, during adolescence. In the case of children and adolescents with high ability, Palazuelo et al. (2010) similarly observe more emotional signs in girls than in boys. However, authors like Prieto et al. (2008) point out that there are no differences in emotional intelligence between high ability and non-high ability students, and no sex differences were observed. In the case of pre-teens (11–13 years old), Cova et al. (2005) observe that women show higher emotional distress and depressive symptoms than males. This coincides with Madden et al. (2000), Khamis (2018), and Van der Cruijsen et al. (2019), who describe that adolescent women refer more emotional distress like anxiety and depression. More research is required on these emotional aspects to confirm these results.

Children and adolescents are at risk of developing mental health problems such as stress, anxiety, and depression before a traumatic event, even when their caregivers respond sensitively, in a receptive and willing manner to their concerns and questions (Bartlett et al., 2020). Although there is evidence that children aged 0–14 years are at lower risk of infection than older children (Zhang et al., 2020), and the physical effects of COVID-19 disease in children have been less than in adults or are generally asymptomatic (Xu et al., 2020), the effects on mental health can increase in a situation like the pandemic. We then must ask ourselves, what happens to them in a situation as exceptional as the pandemic?

Enmienda et al. (2020) say that the current conditions of confinement brought about by safety measures such as social distancing, staying at home, and online schooling can increase, among others, the anxiety and/or sadness in children with high ability. Although they can handle information about the virus cognitively, they can show intense feelings of fear for their family, for themselves, or problems arising from the pandemic (i.e., economic struggles or parents becoming unemployed). Children observe their parent’s behavior and if they see them anxious or worried they can imitate that behavior; however, Boonstra (2020) points out that this anxiety is similar to people could show more generally.

Uncertainty about the implications and the personal effects of COVID-19 creates concern in adults (Brooks et al., 2020), in addition to the psychological effects of the quarantine. As a consequence of their concern, adults may not recognize and respond sensitively to the signs of distress in children in general (Dalton et al., 2020), and particularly in children with high ability in particular (Enmienda et al., 2020). Anxiety can cause children to avoid sharing what worries them as a way to protect others, leading them to confront these feelings alone (Dalton et al., 2020), or leading to the intensification of emotions in the case of children and adolescents with high ability, as they need to be cognitively challenged (Boonstra, 2020).

As has been said before, children are active observers and participants of their environment, and they are not indifferent to the impact of the current health crisis, they feel and react to or simulate adult’s reactions, such as anxiety and stress (Weaver and Wiener, 2020). It is important to consider that, although all children are active observers of the situation and were prone to emotional problems even before the contingency measures were implemented, children with high abilities have additional layers of social and emotional complexity. Some of the most reliable empirical findings about the social and emotional traits of students with high ability have tried to explain that they are more intense. For example, Terman and Hollingworth’s first research in the early twentieth century suggested that students with high ability experience emotional phenomena more deeply than their peers, and call these concerns “gifted intensity” (Wiley, 2020). Some research questions come out of this, including what emotions and reactions do children and adolescents show from the confinement of COVID-19? Are emotions and reactions different between children and adolescents with high ability and with other young adults? Are there differences between age groups and sex?

This work aims to study the emotional reactions of children, with high ability using community samples, to understand their emotions and reactions to the confinement that has been required since the health alert for COVID-19. These results will enable us to develop intervention strategies for this group of people during and after the contingency measures, clarifying debates about the emotional differences between both groups.

## Materials and Methods

### Participants

The sample consisted of 649 children aged between 5 and 14 years old (average = 9.6, ST = 2.6). Table 1 shows data relating to age (kinder: between 5 and 7 years old; children: between 8 and 12 years old, and adolescents: for 13 years and older), sex, and sense of belonging to the community or high ability. Of the sample 627 live in Mexico, 20 live in Spain, and two live in the United States. The selection of the sample was by convenience. The sample of parents with high ability children was obtained through contact with high ability groups belonging to university or civil association networks.

**TABLE 1:** Sex, age, and sample type.

		Male	Female	Total
**High ability sample**				
Age	Kinder	59	38	97
	Children	117	55	172
	Adolescents	28	22	50
**Community sample**				
Age	Kinder	65	45	110
	Children	76	64	140
	Adolescents	34	46	80
	Total	379	270	649

### Measures

A research instrument of 46 open and closed-ended questions was designed, distributed in three sections, namely:

(1) General data, where we explore age, sex, type of school he usually attends, enrollment in psychologic programs, and place of residence.
(2) Knowledge and attitudes toward health contingencies due to COVID-19. Specifically, what they know about the virus, precaution measures, the child’s perception of the state of mind of the adults with whom they live, and also things such as whether they like or dislike staying at home (one of the measures implemented to mitigate the effects of COVID-19).
(3) The emotional reactions shown by children and adolescents in dealing with the COVID-19.

For participants aged 8–14 years old we also used:

• *The Children’s Positive Emotions Questionnaire* (CEIP by its Spanish acronym) (Oros, 2014). This uses a scale that consists of 23 items in a Likert-like structure of 3 points, where the experience of satisfaction, gratitude, joy, serenity, and sympathy for others is explored. For this research, its use was extended up to participants aged 14 years.
• *The PANAS Scales of Positive and Negative Affect for children* (PANASN by its Spanish acronym) (Sandin, 2003), which aims to obtain a measure of both positive and negative effects. For this study, the negative effect scale was used. This negative effect subscale included 10 elements on a three-point Likert-type scale (1, never, 2, sometimes, and 3 many times), which assesses the frequency with which participants experienced negative emotions such as overwhelming fear or tension.

Both questionnaires were authorized by the authors to use in this research.

The goal of the study was presented at the beginning of the questionnaire. In terms of the confidentiality of the data, we asked the parents of participants to indicate whether they consent to the child’s participation in the study.

The reliability of the instruments was established through Cronbach’s alpha, obtaining 0.655 for the scale of the emotional reaction to COVID-19, a positive emotion scale of CEIP, and 0.623 on negative emotions of PANAS.

### Procedure

The study used a Mixed Methods Research focus, combining surveys and cross design, with a qualitative methodology.

The questionnaires were designed with Google formats and distributed to parents through social media (WhatsApp). The instrument contained open questions that were analyzed through the ALCESTE program. The information collected meets the consistency criterion in the texts that correspond to the answers to the open questions and with the criterion of a sufficient amount of information (La société IMAGE, 2015).

This work was conducted ethically according to the principles of the Declaration of Helsinki. The questionnaire contained an informed consent statement at the beginning of the survey, which informed parents about the objectives of the study and other ethical details. The UDG ethics committee authorized the research under the certificate registration number: CI-03820. Data were confidential and we avoided recording information that would allow individual identification of participants.

### Data Analysis

To determine the reliability of the instruments, Cronbach’s alpha test was calculated. To know the relation between variables, Pearson correlation was calculated. To check the relation of the studied variables based on high ability and gender, MANOVA tests were calculated, using the SPSS software v.21.

For the qualitative analysis, we used the ALCESTE software (Reinert, 2001). El Analyse Lexicale par Contexte d’un Ensemble de Segments de Texte (ALCESTE) has been developed to respond to the needs and problems of social researchers when using instruments with open questions, in-depth interviews, or answers (texts) based on projective techniques. This practical software allows the user to statistically analyze a series of words that compose a discourse. It is based on the distribution of the vocabulary, establishing the semantic world of a textual corpus, and examining the structure of the distribution of this corpus through three stages: the construction of the data matrix, the classification of the context units, and the description of the sentences (Gil et al., 1994; De Alba, 2004).

The program identifies the co-occurrence of words in the same sentence, which enables the identification of semantic fields, represented in dendrograms (De Alba, 2004). The main advantage of this software is that the program establishes categories through the analysis it performs (Illia et al., 2012). ALCESTE has been widely used in high capacity studies (Villatte et al., 2011; Rodríguez-Naveiras et al., 2019), the study of COVID-19 representations in children (Idoiaga et al., 2020), and studies on episodic memory and self-awareness in Asperger Syndrome (Chaput et al., 2013), among others.

## Results

### Quantitative Analysis

#### Variable Relation Between Emotions (Positive and Negative) and Reaction to COVID-19

First, the correlation between the three variables was found, which included factors corresponding to positive emotions (Joy, Serenity, Sympathy, Personal Satisfaction) with negative emotions, as well as with the scale of emotional reactions to COVID-19. A subsample of ages between 8 and 14 years old was used (see Table 2).

**TABLE 2:** Correlations among variables.

	Joy	Serenity	Sympathy	Satisfaction	Negative emotions
Serenity	0.737***	1			
Sympathy	0.318***	0.236***	1		
Satisfaction	0.542***	0.546***	0.364***	1	
Negative Emotions	−0.325***	−0.297***	0.110**	−0.218***	1
COVID-19	−0.013	−0.030	0.119**	0.002	0.023

**p < 0.01. ***p < 0.001.

As shown, the positive emotions correlate significantly with each other. With negative emotions, the relation is negative but in Sympathy. With the scale of emotional reactions to COVID-19, Sympathy was the only one to correlate significantly.

#### Differences Between Positive and Negative Emotions

To establish the differences between the high ability and community samples, sex, and age, which are detailed below, data of the subsample of ages between 8 and 14 years old were analyzed.

##### Comparison between student ability (high ability and community samples) and sex

Given there were relations between positive and negative emotions, a MANOVA test was performed with two factors: ability (high abilities/community sample) and sex, with the 8+ years old sample. The descriptive statistics (means and standard deviations) are shown in Table 3.

**TABLE 3:** Descriptive statistics of ability and sex.

		High ability			Community		
		Mean	ST	*N*	Mean	ST	*N*
Negative emotion	Male	9.34	1.67	145	9.21	1.65	110
	Female	9.43	1.79	70	9.34	1.70	110
Happiness	Male	26.39	2.75	145	26.73	2.44	110
	Female	26.45	2.89	70	26.72	2.57	110
Serenity	Male	14.78	1.97	145	14.90	1.93	110
	Female	14.73	2.19	70	15.01	1.89	110
Friendliness	Male	9.20	1.49	145	9.24	1.54	110
	Female	9.61	1.54	70	9.77	1.54	110
Satisfaction	Male	7.36	1.15	145	7.49	1.11	110
	Female	7.58	1.17	70	7.69	1.02	110

The MANOVA is not significant even for the interaction of sex by ability (Wilks’ λ = 0.998, *F*_5,434_ = 0.365, *p* = 0.963, η^2^ partial = 0.002), not even for ability (Wilks’ λ = 0.996, *F*_5,434_ = 0.365, *p* = 0.873, η^2^ partial = 0.004). Nor are there any differences in any of the scales analyzed. In contrast, it is significant for the sex variable (Wilks’ λ = 0.970, *F*_5,434_ = 2.700, *p* = 0.020, η^2^ partial = 0.030), with a small effect size. Women result in significantly higher scores for *Friendliness* (*F*_1,438_ = 10.185, *p* = 0.002, η^2^ partial = 0.023), with large effect size and *Satisfaction* (*F*_1,438_ = 3.856, *p* = 0.050, η^2^ partial = 0.009), but the size of the effect is very small.

##### Differences according to age

To know if the age could be relevant in differentiating between these variables, they were split according to age into two groups: 8–11 (children) and 12–14 (adolescents). Contrasts of *t* Student for all the variables under study were calculated, except in the Joy scale for it being heteroscedastic the Welch solution was used (see Table 4).

**TABLE 4:** Differences according to age.

		*N*	Mean	ST	*t*	fd	*p*	*d*
Negative emotion	Children	312	9.43	1.67	2.174	440	0.030	0.25
	Adolescents	130	9.05	1.69				
Happiness	Children	312	26.61	2.59	0.565	226.42	0.572	0.07
	Adolescents	130	26.45	2.79				
Serenity	Children	312	14.84	1.93	−0.239	440	0.811	−0.02
	Adolescents	130	14.89	2.09				
Friendliness	Children	312	9.48	1.54	1.361	440	0.174	0.13
	Adolescents	130	9.26	1.49				
Satisfaction	Children	312	7.51	1.11	0.165	440	0.869	0.01
	Adolescents	130	7.50	1.10				

##### Differences in the response scale to COVID-19

All the sample results were used in the analysis (participants between 5 and 14 years old), thus, adding an age group (Kinder, 5–7 years old). The results of average and standard deviations, per ability, sex, and age are shown in Table 5.

**TABLE 5:** Descriptive statistics of ability and sex and age.

		High abilities			Community		
		Mean	ST	*N*	Mean	ST	*N*
Kinder	Male	2.44	0.74	59	2.25	0.85	65
	Female	2.39	0.75	38	2.56	0.77	45
Children	Male	2.21	0.88	117	2.20	0.80	76
	Female	2.42	0.81	55	2.39	0.79	64
Adolescents	Male	2.00	0.90	28	1.76	0.89	34
	Female	2.27	0.88	22	2.46	0.78	46

The ANOVA of ability was calculated for sex and age, as shown in Table 6. There are differences in sex, with a small size effect where females are more concerned; and in age, also with a small size effect where the significant differences between children and adolescents (Tukey’ HDS = −0.25, *p* = 0.017), and the youngest show more concern.

**TABLE 6:** ANOVA differences by ability, sex, age, in concern for COVID-19.

Contrast	*F*	*p*	η ^2^ partial
Full interaction	1.102	0.333	0.003
Age vs ability	0.001	0.999	0.001
Sex vs ability	3.264	0.071	0.005
Age vs sex	1.859	0.157	0.006
Ability	0.093	0.763	0.001
Sex	14.813	0.001	0.023
Age	4.617	0.010	0.014

### Qualitative Analysis

The data from the four open-ended questions of the study were analyzed, distinguishing the two samples of the research: high ability and community samples. These results are discussed below. An Elementary Context Unit (ECU) is a unit of text within which ALCESTE calculates the frequency of word co-occurrences (Reinert, 2001).

First question: “I understand I have to stay at home because…”

**FIGURE 1:**
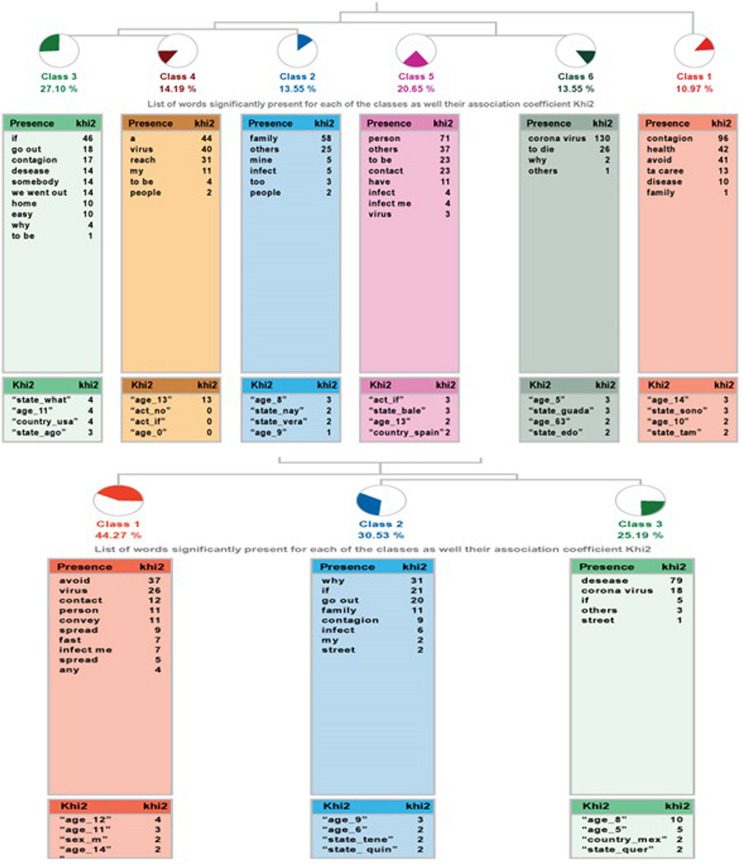
Dendrogram of “I understand that I have to stay home because…,” community sample at the **(top)**, high-capacity sample at the **(bottom)**.

The ALCESTE analysis for the question *“I understand I have to stay at home because…”* in which the community sample yielded six classes, classifying 48% of the wording units. It is organized as follows: class 1 connects with two branches, a set formed by classes 5 and 6, and a second branch, where class 2 connects with 3 and 4 (see the left side of Figure 1).

With the sample of high ability students, the percentage of the corpus groups analyzed is 42%. In this case, the answers are grouped into three classes, where the first one links to a grouping between classes 2 and 3, as can be seen on the right side of Figure 1.

Table 7 presents more detail relating to each class. These include name, number of ECUs, percentage of variable been explained, the most representative word, and example sentences for each class. In the community sample, class 1 has a more general component, while class 2 and the branch that derive from it with classes 3 and 4 talk about the contagion in terms of the possibility that it might infect others and the danger posed by the contagion. The other two classes, 5 and 6, refer to isolation and the need to avoid contagion and the lethal risks of the infection. In the sample of the participants with high ability, answers are more concentrated.

**TABLE 7:** Information on the analysis of the question “I understand I have to stay at home because….”

Class		ECUs	%	Word
**Community sample**				
1 Name	*Avoid contagion*	17	10.97	Contagion
Phrases	It is a highly contagious disease This way the risk of spreading infection is reduced, slowing it down and avoiding an overload of the country’s health system. I must avoid contagion			
2 Name	*Don’t infect me nor others*	21	13.55	family
Phrases	Yes, my safety and others that I live with, including my family To not infect me or infect others I can catch the disease and pass it to my family			
3 Name	*Don’t go out to avoid contagion*	42	27.10	If
Phrases	Because if I go out someone could infect me Because if we go to the streets someone with the disease could infect us. Because if I go out I can get sick.			
4 Name	*Dangers of contagion*	22	14.19	One
Phrases	If I catch the virus I could pass it to my parents and we could have a serious problem. It’s a very contagious virus. I can be the carrier of the virus and pass it along to others.			
5 Name	*Social isolation*	32	20.65	Person
Phrases	Because we need to avoid contact with other people that could be infected. I can get infected by being with other people. Because by being in touch with other people you are more likely to get sick.			
6 Name	*Coronavirus infection*	21	13.54	Coronavirus
Phrases	I could get coronavirus and I could die. Because I could get infected with coronavirus and I could die. To not get infected by the coronavirus.			
**High ability sample**				
1 Name	*Avoid spreading the virus*	58	44.27	avoid
Phrases	To avoid the spread of the virus This way I avoid spreading the virus It is the best way not to spread the virus			
2 Name	*Chance of contagion*	40	30.53	because
Phrases	Because if I go out I will get infected. It’s possible that nothing may happen to me, but if I get the virus, I’m a carrier and I could pass it along to my family. Because if not I could get infected.			
3 Name	*Chance of getting sick*	33	25.20	Get sick
Phrases	I could get sick with coronavirus COVID_19 Due to the coronavirus, if we go out we might get sick I could get sick.			

Comparing the results between both samples, we observed a greater dispersion among children in the community sample, as explained above. While the responses of the high ability group are focused on avoiding the spread of the virus, with two approaches: to become infected (without showing the effect this implies), or to become ill (which implies this infection)

Second question: “What I like the most of staying home is…”

**FIGURE 2:**
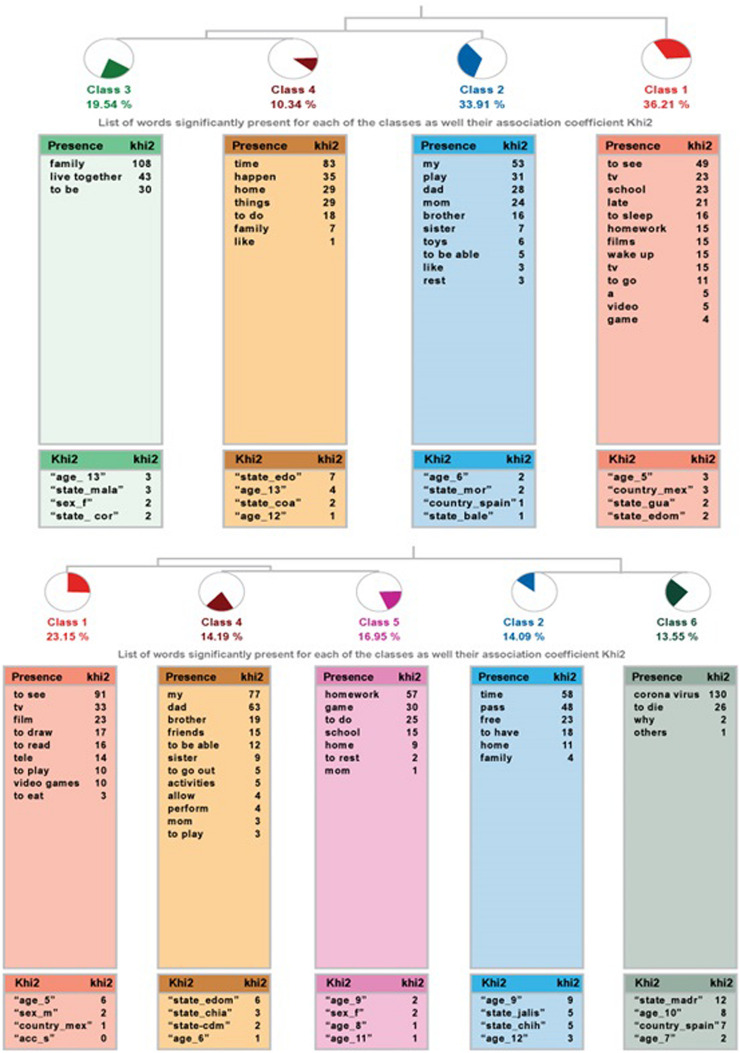
Dendrogram of “What I like most about staying home is…” community sample **(top)** and high-capacity sample **(bottom)**.

The ALCESTE analysis for the question *“What I like the most of staying home is…”* in the community sample led to four classes, classifying 54% of the wording units. Class 1 branches into a structure that links class 2 and a final branch to classes 3 and 4 (see Figure 2, left).

For the sample of students with high abilities, there are five classes, which include 56% of the wording units. As displayed on the right side of Figure 2, there are two groups: on the one side, class 1, which links jointly with classes 4 and 5. The other branch gathers classes 2 and 3.

Table 8 shows the classes in terms of name, number of grouped ECUs, the percentage of the corpus that it explains, and the most representative word, as well as the example phrases of each class. In the community sample, the most relevant class, considering the number of ECUs it incorporates is class 1, *Watch TV*. In contrast to activities more centered on personal focus, like watching TV or playing, the last two classes seem to focus more on social aspects (being with the family) or widening to more open interests.

**TABLE 8:** Information on the analysis of the question “What I like the most about staying home is…” students of community sample and the high ability sample.

Class		ECUs	%	Word
**Community sample**				
1 Name	*Watch TV*	63	32.21	Watch
Phrases	Watch TV and sleep a little more Watch TV, watch videos I don’t wake up early to go to school and also watch TV.			
2 Name	*Chance to play*	59	39.59	My
Phrases	Play, rest, help my parents I can play with my friends through videocall I can play with my sisters			
3 Name	*Being in family*	34	19.54	Family
Phrases	Being with my family Being with my family and enjoyment Interact with my family.			
4 Name	*Chance of doing other things*	18	10.34	Time
Phrases	I can take advantage and can do many things that I couldn’t do before due to lack of time. I’m safe at home and it is not a big inconvenience, just washing the hands all the time. That I can do more things freely.			
**High ability sample**				
1 Name	*Watch TV*	41	23.16	Watch
Phrases	Watch TV read and play Watch TV and play I can read and play videogames			
2 Name	*Free time*	26	14.69	Time
Phrases	That I have more free time and I like working at home better That I have free time Having more time for me			
3 Name	*Being with family*	44	24.86	Family
Phrases	That I wake up a little later and I interact more with my family Being with my family I can be with my family and be safe.			
4 Name	*Chance to play with family*	36	20.34	My
Phrases	Able to do more activities with my parents Able to do activities that enhance my abilities and skills. Play with my little sister, with my older brothers, and with my parents			
5 Name	*Doing school homework*	30	16.95	Homework
Phrases	Do school homework I play more and do my homework with ease To rest and to do homework			

In the high ability sample, the class with more grouped ECUs is class 3, followed by class 1. The first class is linked to the set of classes 2 and 3, whose contents show deeper interests (free time, being with family). The other branch is made by classes 4 and 5, and is more focused on activities: playing or doing homework.

A comparison of both samples shows that the answers are very similar, except for the fact that the students with high abilities indicate that they perceived school homework as something positive during their obligatory stay at home.

Third question: “What I dislike the most about staying home is…”

In the ALCESTE analysis for the question*: “What I dislike the most about staying home is…”* the community sample yielded five classes, classifying 54% of the wording units. There are two branches: one that connects classes 4 and 5, and that one that links class 1 to 2 and 3.

The high ability sample shows the most relevant analysis of this research since it allows us to classify 70% of the wording units. Class 1 links the other two that are grouped.

**FIGURE 3:**
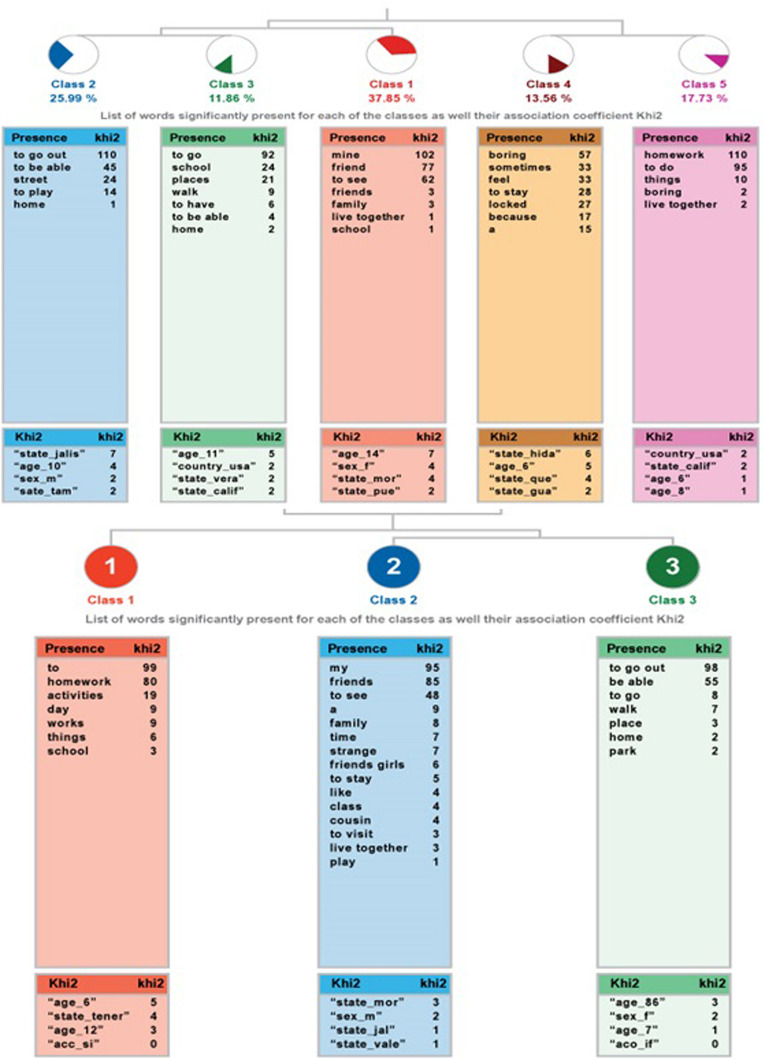
Dendrogram of the analysis to the question “What I dislike the most about staying home is…” community sample **(top)** and high ability sample **(bottom)**.

Both dendrograms can be seen in Figure 3.

Table 9 shows the specifications of the classes, in terms of name, the ECUs that are included, the percentage of variable explained, the most representative word, and example sentences for each class. In the community sample, the most representative class is the 1, which groups more ECUs. As opposed to the link that has to do with boredom (class 4) and homework (class 5), the other set of classes focuses on what it is not allowed to do by staying at home: seeing friends, going out to the street, or other places.

**TABLE 9:** Information on the analysis of the question “What I dislike the most about staying home is…” community and high abilities samples.

Class		ECUs	%	Word
**Community sample**				
1 Name	*Impossibility of seeing my friends*	67	37.85	My
Phrases	I cannot see my friends I don’t see my friends nor family Not seeing my Friends			
2 Name	*Impossibility of going out to the street*	46	25.99	Go out
Phrases	Not able to go out to play I’m not allowed to go out to the street Not able to go out to the Street			
3 Name	*Impossibility of going places*	21	24.86	Go
Phrases	I cannot go to places I like Not going to school Not able to go to museums or visit places that I would like to know			
4 Name	*Boredom*	24	13.56	Get bored
Phrases	It’s because I get bored a little Being locked down and bored That I’m bored sometimes			
5 Name	*Doing school homework*	19	10.74	Homework
Phrases	Doing homework Doing the homework they sent me It is a homework-fest for teachers, we do much more of what we do normally in class, and when I don’t understand there is no one to explain them to me			
**High ability sample**				
1 Name	*Have to do homework*	53	24.09	Do
Frases	That they give us school homework That I have to do homework That I have to do homework every morning			
2 Name	*Impossibility of seeing my friends*	106	48.18	my
Phrases	That I lose track of time and I don’t see my friends Not seeing my friends and family That I can’t see my friends			
3 Name	*Impossibility of going places*	61	27.73	Go out
Phrases	Not able to go to other places Not able to go out to fun places Not able to go out			

In the sample of high abilities, the class that groups most units is the impossibility of seeing friends, in this case, class 2. As in the previous case, but more concentrated, there are two connections: the general in class 1, where that it bothers them to do homework, as opposed to the impossibility of both going out (class 3) and seeing friends.

Despite being broken into more classes, both samples share two general categories: what they have and can do and what is not allowed. Both samples feel upset about school homework and because they are not allowed to go out to see someone or go out wherever they like. On the other hand, students from the community sample show boredom as something that bothers them, but this is not the case with the high abilities sample.

It is important to highlight that in the previous question, high ability students were more likely to do homework, while in this question they dislike it. A possible explanation is that they could do school work at their own pace, instead of feeling forced to do it.

Fourth question: “Do you want to add something else?”

The ALCESTE analysis for the question *“Do you want to add something else?”* in the community sample has yielded three classes, classifying 61% of the wording units. There are two branches, one that links class 1 with the other two, which are also connected in between.

For the high ability sample, this question portrays the highest number of classes of the questionnaire: seven, which points to a higher diversity in the information to be provided. It allows us to classify 50% of the content. As in the previous analysis, class 1 works as a link for the others, connecting each other by pairs: classes 2 and 3, 4 and 5, as well as 6 and 7.

**FIGURE 4:**
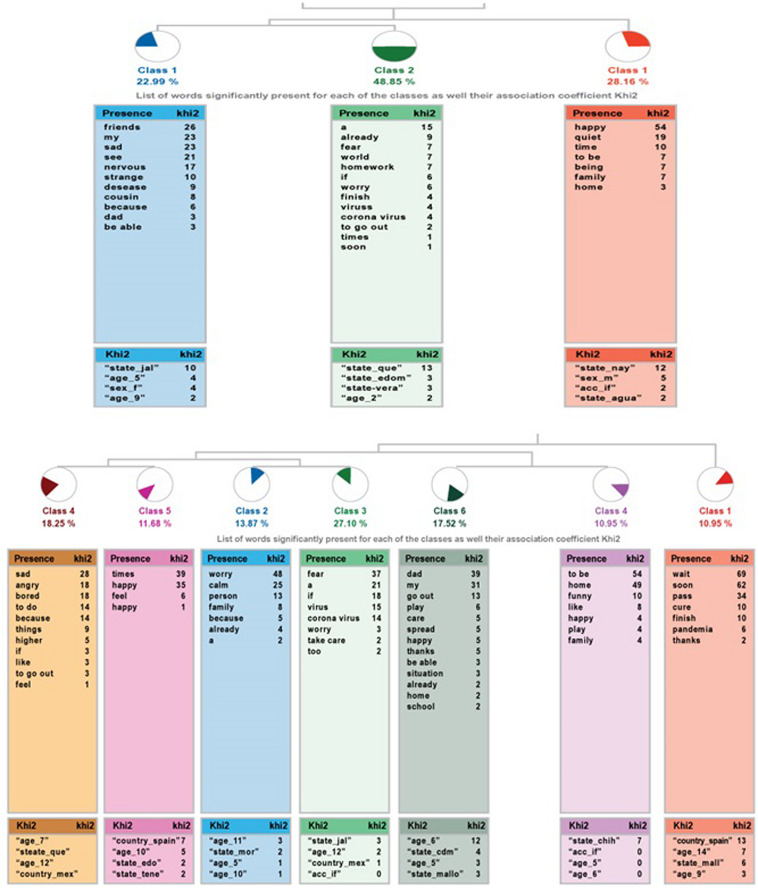
Dendrogram of the analysis to the question “Do you want to add something else?,” community sample **(top)** and high ability sample **(bottom)**.

Figure 4 shows the dendrograms of the community sample (left side) and the high ability sample (right side).

In the community sample (Table 10), the contents have two different approaches: class 1 experience joy from being at home. The other two classes have a negative component: either sadness or concern, the most relevant class in terms of the ECUs grouped.

**TABLE 10:** Information on the analysis of the question “Do you want to add something else?” community and high abilities samples.

**Class**		**ECUs**	**%**	**Word**
**Community sample**				
1 Name	*Happy about being home*	49	28.16	happy
Phrases	I feel happy about spending more time with my family I feel happy about being with my family I feel happy			
2 Name	*Sadness*	40	22.99	friends
Phrases	I feel sad because I can’t see my friends and cousins Sad because I don’t see my cousins Sad because I can’t share with my friends			
3 Name	*Concern*	85	48.85	a
Phrases	A virus that we must not fear with all the fear of the world but we have to be informed and take care to not exaggerate, well, that’s all I want the COVID-19 pandemic to be over Good, I am a little worried			
**High ability sample**				
1 Name	*Hope*	15	10.95	Hope
Phrases	I hope that the pandemic ends soon and everything goes back to normal I am concerned about all that is happening and I hope that the quarantine ends soon I hope that this pandemic ends soon and I hope that many will be alive to enjoy it			
2 Name	*Concern*	19	13.87	Concern
Phrases	Calmed and at the same time concerned Calmed and a little concerned What I feel is concerned because I want to be with my family for a while so that I can see my family and my best friend called Karen			
3 Name	*Fear*	23	16.79	Fear
Phrases	A synthetic virus and yet we’re still healthy and if it gets to us the coronavirus doesn’t mean we will die in lockdown I feel good because I’m at home, I fear coming back to school. If we are sick use a mask and let our parents know in case we feel bad so they can take us to the doctor. I feel a little concerned because this Chinese pandemic is spreading too fast and I fear that the coronavirus will get to me. Also if I get sick I think I will go to China to get cured.			
4 Name	*Sadness*	25	18.25	Sad
Phrases	I feel sad and upset because I can’t do my daily things I’m sad because we’re not going on vacation I’m sad because my grandfather died			
5 Name	*Joy*	16	11.68	Times
Phrases	I feel happy Sometimes I feel excited I feel happy to have a great family			
6 Name	*Care to avoid getting infected*	24	17.52	Parents
Phrases	Do not get the infection, being with my parents, not able to go out We must take care to not get infected, play with my parents Calmed because I know that my parents will take care of me			
7 Name	*Being comfortable at home*	15	10.94	Being
Phrases	It’s very fun to be at home I like being at home I feel that being at home is fun			

In the high ability sample, there is more dispersion, as seen by the seven classes obtained. Classes 2 and 3 are connected due to their negative nature: concern and fear. In contrast, classes 4 and 5 have opposite emotions: sadness (being the class in which most ECUs are grouped) and joy. The other two, group the family and home safety against the virus.

In the comparison between both samples, it is observed that in response to a completely open-ended question (“Do you want to add something else?”) the high ability group shows many more variations. Both groups swing on the dichotomy of the negative and positive sides, but the higher abilities’ group also integrates a safety perspective of being protected by the family. Moreover, children from the community sample show higher percentages of concern and sadness than those with higher abilities.

## Discussion

It is important to highlight that the tool designed to analyze the concern generated by the pandemic is not linked to emotions that are positive or negative. The insights shown by this tool, which has sentences starting with wording like *“I feel…*,*”* could have a more rational component rather than emotional.

Both age and sex have significant differences. Female students show more concern, but also more sympathy and personal satisfaction, which is in line with findings in the literature which included emotional scores higher to female target, although the effect size findings are small. They are also more concerned but the effect is small (Sharp et al., 2006; Van der Graaff et al., 2018).

In terms of age, there are some differences in both negative emotions and concern about COVID-19. The odd thing is that, according to the results, adolescents are less worried than children, when it could be expected to be the opposite since they have a better knowledge of what is happening, which could also be the reason they think this way.

Comparing the samples by abilities does not allow us to establish the differences between groups on a quantitative level. They do not differ in any of the used tools. These results support research that points out that the students with high abilities differentiate in cognitive aspects, which is obvious, but not in their personal and social adjustment (Borges et al., 2011), neither in their emotional reaction (Valadez et al., 2015; Egeland, 2019; França-Freitas et al., 2019).

This changes when the verbal language given by the minors is analyzed. In two questions, the sample of high abilities is more concise: the reasons given for staying at home seem to be more precise: for example, they involve a general grouping (avoiding contagion) and two more specific reasons, so as not to infect others or infect themselves and what bothers them about staying at home: forced school homework and the impossibility of going out and meet friends, which they share with the community sample. In both groups, the social character stands out and the need to socialize with their peers. In contrast, in the community group, boredom is a class that is not present in the high ability sample. We could make a hypothesis that, by having the freedom to do activities of their own choice, there is no boredom (which is a usual complaint when at school, if it does not meet their educational needs (Pomar and Díaz, 1998; George, 2011), because they have sufficient strategies for looking for entertainment.

In terms of interests and autonomous work, we found an emphasis that only appears within the high abilities sample: when asked what it is what they like the most, “doing homework” is one class. This could seem contradictory (as it is also what they dislike the most) but a possible explanation could be how they perceive homework as something they do at their own pace or that homework is forced, and or that it is not hard enough and does not meet their ability level.

Where the greatest difference is observed, in terms of greater richness and variety of responses, the last question is more open-ended than the previous one. The community sample shows only three classes that are shared with the high ability group (being at home, sadness, and concern) while the other four are only present within the latter: joy, fear, hope, and precautions. The community sample showed more negative feelings than the one with high abilities.

The main limitation of this study is that we used an online questionnaire, which means that there is no guarantee that it was answered by the child without parental influence. This could perhaps be the reason for the youngest group showing more concern because the parents could be sharing their concerns as they feel the need to protect their children.

Additionally, there were other limitations in this study, including the fact that the level of physical activity undertaken by the children was not investigated, so no comparisons can be made between physical activity before and during the pandemic.

The situation created by the pandemic, which has involved an international quarantine, has been a historical moment, as it has forced us to live in unexpected circumstances of great uncertainty. Nevertheless, there does not seem to be a clear emotional disadvantage for the samples presented in this study at the time it was conducted (March and April 2020). It is, therefore, necessary to continue investigating these emotional states as these circumstances become the new normal, and it is important to understand the first-hand experiences of children who have lived through this unusual period. This allows us to understand and deepen insights into the effects of lockdowns and social isolation and the effects these can have on children and adolescent populations, but also to learn and seek intervention and coping strategies if a similar situation occurs again in the future.

## Data Availability Statement

The raw data supporting the conclusions of this article will be made available by the authors, without undue reservation.

## Ethics Statement

The studies involving human participants were reviewed and approved by the Research Ethics Committee of University of Guadalajara, registered with the National Commission on Bioethics with number CONBIOÉTICA-14-CEI-002-20191003. OPINION of manuscript CI-03820. Written informed consent to participate in this study was provided by the participants’ legal guardian/next of kin.

## Author Contributions

MV and GL-A contributed to the conception and design of the study. MV, GL-A, NR, GO, FF, and CR designed the questionnaire. GO and FF organized the databases. ÁB performed the statistical and qualitative analysis. NR, MV, FF, and GL-A wrote the introduction. MV, ÁB, and GL-A wrote sections of the manuscript. All authors were involved in the collection of the data, contributed to manuscript revision, and read and approved the final version.

## Conflict of Interest

The authors declare that the research was conducted in the absence of any commercial or financial relationships that could be construed as a potential conflict of interest.
